# Advances in Total Hip Arthroplasty: A Comprehensive Review of Surgical Techniques, Implant Designs, and Postoperative Outcomes

**DOI:** 10.7759/cureus.110216

**Published:** 2026-06-03

**Authors:** Kotteda Anil Kumar, Shobhit, Jishnu Jonnalagadda, Akshay Sreedharan, Rajiv N Daveshwar, Ashwin M Sathe

**Affiliations:** 1 Department of Orthopaedics, All India Institute of Medical Sciences, Mangalagiri, Mangalagiri, IND; 2 Department of Orthopaedics, District Male Hospital, Moradabad, IND; 3 Department of Orthopaedics, Balaji Institute of Surgery, Research and Rehabilitation for the Disabled (BIRRD) Hospital, Tirupati, IND; 4 Department of Orthopaedics, All India Institute of Medical Sciences, Kalyani, Kalyani, IND; 5 Department of Orthopaedics, Dr. N D Desai Faculty of Medical Science and Research, Dharmsinh Desai University, Nadiad, IND; 6 Department of Orthopaedics, Jupiter Hospital, Pune, IND

**Keywords:** implant fixation, perioperative recovery, surface design, surgical approach, total hip arthroplasty

## Abstract

Total hip arthroplasty (THA) is a key surgical procedure for the treatment of end-stage hip disease and a primary tool for restoring mobility and quality of life globally. Although the field is evolving rapidly, evidence about advances in THA is sparse and scattered over different surgical techniques, implant designs, and perioperative strategies, making it difficult to interpret at a system level. This review aims to unite and synthesize the current evidence critically to understand the impact of interacting technical and biological factors on durable outcomes. PubMed, Embase, Web of Science, and the Cochrane Library were searched from 2015 to 2025 for an integrative review of the narrative. Randomized trials, comparative observational studies, registry analyses, and high-quality systematic reviews of primary THA were included. The evidence synthesis included the focus on the mechanistic connections between component positioning, fixation biology, bearing performance, and recovery environments with functional durability and implant survivability. Key findings suggest that no single innovation is enough to ensure success, and coordinated optimization of the accuracy of alignment, soft-tissue management, fixation strategy, and improved patient recovery pathways will consistently impact stability, wear characteristics, and patient-reported performance. It is often difficult to distinguish the relevance of methods and endpoints that are not the same. Integrated evaluation helps guide patient-specific treatment decisions, technology selection, and innovation that aligns with value-based care. This review firmly establishes that the engineering approach to viewing THA as a clinical system provides a clearer understanding of advances and direction of future investigations.

## Introduction and background

Total hip arthroplasty (THA) is one of the most successful and widely performed reconstructive procedures in modern medicine to treat end-stage hip disease and regain mobility and quality of life for millions of patients throughout the world each year [1]. Alongside these benefits, modular implant designs have introduced specific mechanical complications, including trunnionosis, which refers to corrosion and wear at the femoral head-neck junction and may contribute to adverse local tissue reactions or implant failure [2]. Global use is still rising with the aging population, expansion of indications to younger and fitter patients, and dependence on arthroplasty by health systems to cut the socioeconomic burden of disability [3]. Evidence has been shown of sustained increases in the volume of procedures performed with increased expectations of durability, quick recovery, and patient-reported satisfaction [3]. This increase in demand has led to an increase in the scrutiny of the performance of surgery and the longevity and value-based outcomes of implants in a wide variety of patient populations and care environments [4]. The theoretical basis of THA is based on the following: biomechanical restoration, biologic fixation, and tribological optimization, meaning the reduction of friction, wear, and debris generation at the artificial joint surface, aiming to recreate stable, low-friction articulation under physiologic loading conditions [5]. Successful results require accurate planes of the restoration of the hip center, offset, and leg length with secure fixation that allows for long-term transfer of loads without causing stress shielding and micro-motion-related failure [6].

Bearing surface selection and head/neck junction design are involved in the generation of wear, the biology of particulate exposure, and the inflammatory mechanisms that are the basis for osteolysis, or progressive bone loss caused by inflammatory responses to wear debris, and late aseptic loosening [7]. Soft-tissue handling, capsular integrity, and spinopelvic kinematics provide additional modulation of joint stability, showing that mechanical success is not the consequence of a single aspect of joint design or execution but rather the result of coordinated biomechanical, implant-related, and soft-tissue factors [8].

There are several surgical techniques, such as posterior, direct anterior, and anterolateral approaches, each with its own pros and cons in terms of exposure, soft-tissue preservation, and complication rates [9]. Preoperative planning and digital templating, navigation devices, and robotic assistance have been focused on minimizing variation in component positioning and maximizing desired biomechanical goals to increase reproducibility [10]. The technology of implants itself has experienced parallel innovation with the introduction of better porous cementless femoral surfaces, improved stem shapes, better acetabular modular systems, and longer bearing couples such as hydroxyapatite-coated metal stems with highly crosslinked polyethene liners [11]. The other aspect of perioperative care that has evolved is improved recovery pathways, multi-modal analgesia, blood conservation, and outpatient protocols to improve recovery while maintaining safety [12].

Despite these developments, there is still fragmentation of the literature evaluating advances in THA, with large heterogeneity in methodology that allows no definitive conclusions about comparative effectiveness [13]. Short-term surrogate markers such as pain scores or length of stay are typically reported, while longer-term clinically meaningful outcomes such as stability, long-term function, and revision are only evident over the long term [14]. There is evidence that survivorship data from observational cohorts and large databases are confounded by patient selection, surgeon experience, and institutional practice patterns [15]. Innovations are frequently evaluated in isolation, and results can be driven by the combination of surgical approach, implant, and postoperative management, which can either positively or negatively influence the outcome [16].

Another drawback is that there is still inconsistent reporting of patient-reported outcome measures and of what constitutes a clinically meaningful change on the measure [17], and the synthesis across studies is difficult. Despite the growing prevalence of these subgroups, and the disproportionate risk and burden of complications, they are treated differently: obesity, osteoporosis, spinopelvic imbalance, and fracture-related indications [18]. New failure modes, such as periprosthetic fracture, trunnionosis, and infection, suggest that progress in one failure mode could be offset by the introduction of new failure modes with changes to design and/or technique [19]. This lack of information underscores the need for critical and integrated evaluation, which helps to combine the knowledge from surgery techniques, principles of implant design, and the approach of postoperative management in a conceptual framework [20]. This type of approach is effective and allows a correlation between the improvement over time of the accuracy of the mechanism and its biological fixation, and the improvement in clinical situations in terms of durable and patient-centered results [21,22].

In challenging trade-offs and finding a set of boundary conditions for best application and delineating rational choices for adoption of new technologies, the goal of this review is to critically analyze the development of THA using the lens of synthesis, rather than a descriptive enumeration of innovations [23-24] that emphasizes mechanism, outcomes, and value. The review focuses on evidence, concordance, relevance, and implications for future research [24]. Accordingly, advances in THA should be interpreted not as isolated improvements in approach, implant, or recovery pathway, but as interdependent changes whose clinical value depends on patient-specific biomechanics, biological response, surgical execution, and postoperative recovery conditions. This perspective aligns with the principles of informed decision-making and innovation’s alignment with patient-centered outcomes [25].

Objectives of the review

This review aims to highlight the critical evaluation of the latest advances in THA and summarize the evidence for surgical techniques, implant designs, and postoperative care pathways for the implant. The focus is placed on the methodological rigour, coherence of mechanisms, and relevance to outcomes to assess whether the innovation reported leads to long-term clinical benefits. The review will draw on an array of heterogeneous evidence to identify patterns of effective practice, areas where there are limitations that need to be addressed, and priority areas where there is a need for future research and clinical optimization.

## Review

Methodology

This review was designed as a comprehensive narrative review of advancements in primary THA, focusing on surgical techniques, implant design, fixation strategies, component positioning, perioperative care, and postoperative outcomes. It was not designed as a systematic review or meta-analysis; therefore, Preferred Reporting Items for Systematic Reviews and Meta-Analyses (PRISMA)-based procedures, including a formal PRISMA flow diagram and quantitative pooling, were not applied. No statistical synthesis was performed; therefore, no meta-analysis, meta-regression, pooled effect estimates, pooled risk ratios, pooled hazard ratios, p-values, or 95% confidence intervals were calculated. This approach was selected because the review aimed to provide a broad mechanism-informed synthesis across heterogeneous clinical, biomechanical, implant-related, and perioperative domains rather than exhaustive study identification or statistical aggregation.

A structured literature search was performed in PubMed/MEDLINE, Embase, Web of Science, and the Cochrane Library. The search covered publications from 2015 to 2026 and was restricted to English-language studies involving human subjects. Representative search terms included combinations of “total hip arthroplasty,” “total hip replacement,” “primary THA,” “surgical approach,” “posterior approach,” “direct anterior approach,” “anterolateral approach,” “implant fixation,” “cemented fixation,” “cementless fixation,” “bearing surface,” “highly cross-linked polyethylene,” “ceramic head,” “dual mobility,” “robotic arthroplasty,” “computer navigation,” “component positioning,” “spinopelvic mobility,” “enhanced recovery,” “outpatient arthroplasty,” and “patient-reported outcomes,” combined using Boolean operators such as AND and OR. MeSH-style concepts included “Arthroplasty, Replacement, Hip,” “Hip Prosthesis,” “Prosthesis Design,” “Postoperative Complications,” “Treatment Outcome,” and “Recovery of Function.” The search was updated during revision to capture recent evidence on additive manufacturing, highly porous or trabecular titanium cementless fixation, technology-assisted arthroplasty, robotic planning, and patient-specific dynamic safe-zone concepts. Reference lists of relevant reviews and key articles were also examined manually to identify additional literature suitable for inclusion in the narrative synthesis.

For transparency, the search initially identified 214 records. After removal of 48 duplicates, 166 records were screened by title and abstract. Of these, 96 records were excluded because they were not directly relevant to primary THA, contemporary implant technology, surgical approach, fixation strategy, perioperative care, or postoperative outcomes. Full-text assessment was performed for 70 articles, and 50 studies were finally included in the synthesis. The 20 full-text exclusions consisted of case reports, small non-comparative series, technical notes without outcome data, studies focused exclusively on revision arthroplasty, and studies evaluating outdated implant technologies.

Eligible literature included randomized controlled trials, comparative observational cohorts, registry-based analyses, and high-quality reviews addressing surgical approaches, implant fixation, bearing characteristics, perioperative pathways, or clinical outcomes following primary THA. Titles, abstracts, and eligible full texts were assessed for relevance by two reviewers, and disagreements were resolved through discussion and consensus. Because this was a comprehensive narrative review rather than a systematic review, formal risk-of-bias scoring was not used as an exclusionary framework. Instead, methodological considerations such as study design, population characteristics, follow-up duration, comparability of groups, outcome reporting, and risk of confounding were considered during interpretation of the evidence. Evidence was weighed narratively according to methodological relevance and clinical applicability. Randomized trials were prioritized for short-term comparative efficacy when available, whereas registry-based studies were considered particularly useful for survivorship, revision risk, and uncommon complications. Systematic reviews and high-quality comparative cohorts were used to identify consistency across the literature. Conflicting findings were interpreted in relation to study design, sample size, follow-up duration, patient selection, surgical expertise, and outcome definition.

Data extraction focused on study design, patient characteristics, intervention characteristics, comparators, follow-up duration, and reported outcomes, including complications, patient-reported metrics, functional recovery, implant survivorship, and revision risk. Findings were synthesized using a comparative and mechanism-informed narrative approach across surgical techniques, implant designs, fixation strategies, component positioning, and perioperative pathways. This method enabled critical appraisal of contemporary THA advances while preserving the intended scope of a comprehensive review.

Epidemiology and evolving indications for total hip arthroplasty

THA has become a mainstay treatment for end-stage hip pathology, and procedure volumes have been steadily increasing worldwide with population aging, increasing prevalence of osteoarthritis, and increasing life expectancy [24]. Large-scale epidemiological analyses show that this growth is no longer restricted to elderly cohorts to a greater extent because the younger, more active patients increasingly undergo arthroplasty with aspirations of long-term durability and a high level of function [25]. This demographic growth has led to the changing of the clinical goal from short-term pain relief to sustained biomechanical function, implant survivorship, and patient-reported quality of life over decades instead of years [26]. Health systems are burdened with increasing accumulations of revision and with increasing economic impact, in which THA is both a clinical success and a long-term resource challenge [27]. From a mechanistic perspective, the growing indications demonstrate a more extensive spectrum of bone quality and activity profiles as well as comorbidity burdens, which amplify the power of outcome to ultimate implant selection, fixation strategy, and execution of the surgery [3]. Evidence has implicated that the failure mechanisms can be vastly different between age groups and indications, with young patients being more susceptible to wear-related and mechanical failures, whereas the older populations are at risk of early complications and fracture [18].

These patterns highlight the crucial role of epidemiology in shaping the dominant biomechanical and biologic stresses over time in implant performance. The majority of the epidemiologic evidence is based on national registers and administrative databases, which provide the ability to capture trends in revisions and mortality, but are generally less granular about surgical technique or alignment target and postoperative care pathway [15]. Thus, associations of demographic shift with outcome are not always interpreted without consideration of interactions with other variables, such as approach selection, fixation method, and/or recovery protocols [9]. This is a limitation that translates trends at the population level to clinical decision-making, which is more challenging [16]. An occurring sunspot is relevant through understanding that changing indications basically overhaul the risk/advantage cost/benefit wholesale of THA, a necessity of adaptive strategies over uniform answers [2]. Subsequently, it is important to be aware of the population in which arthroplasty is offered, the stage of disease, and under what functional demand. Epidemiological trends thus define the ground level on which mechanistic innovation and integrative evaluation can be built to ensure that the advancement of THA is in step with the current patient population and long-term clinical objectives [22].

Biomechanical and biological foundations of hip arthroplasty success

The long-term success of THA is fundamentally dependent on the relationship between biomechanical reconstruction and biologic response at the bone-implant interface, but not by any one of these technical variables alone [28]. Restoration of the hip center of rotation, femoral offset, and limb length controls the joint reaction forces and the efficiency of the abductor muscles and gait mechanics, and is directly related to functional performance and implant loading patterns [29]. Deviations from these biomechanical targets can generate impingement risk, instability, and distribution of uneven stresses that can predispose constructs to failure in a relatively short period despite the technically adequate implantation [7]. Another factor of durability is biologic fixation, particularly in cementless constructs, which requires surface architecture, initial stability, and quality of host bone [30]. A net amount of micromotion beyond physiological levels disrupts the bone and leads to a decrease in ingrowth, instead of fibrous fixation, which creates a non-clinical pathway to aseptic loosening for years [11]. Too rigid constructs may encourage stress shielding and proximal bone loss, demonstrating that the success of fixation is a balance and not merely more rigid [6].

Tribological considerations provide, in addition, an interaction that links biomechanics and biology, such as wear particle formation and local inflammatory cascade processes [31]. Bearing surface interaction, head size, and the accuracy of alignment are the combined factors that are responsible for volumetric wear, whereas the particulate debris can initiate macrophage-mediated osteolysis that compromises long-term fixation [19]. Modern materials such as highly cross-linked polyethene and ceramic heads lower the wear rates, but their clinical advantage depends on the controlled alignment and the correct activity levels of the patient [13]. Methodological interpretation of these mechanisms is confounded, as many of these studies have inferred biologic or biomechanical benefit in terms of surrogate markers such as radiographic alignment or initial migration data [15]. While such measures allow mechanistic insight, they are charged with a relationship with the risk of revision and sustaining functional benefit that is not deterministic but likely and probabilistic [16]. This mismatch is compounded in patients as they can mask the varying responses because of the bone quality, age, or functional requirement of the patient [3].

**Figure 1:**
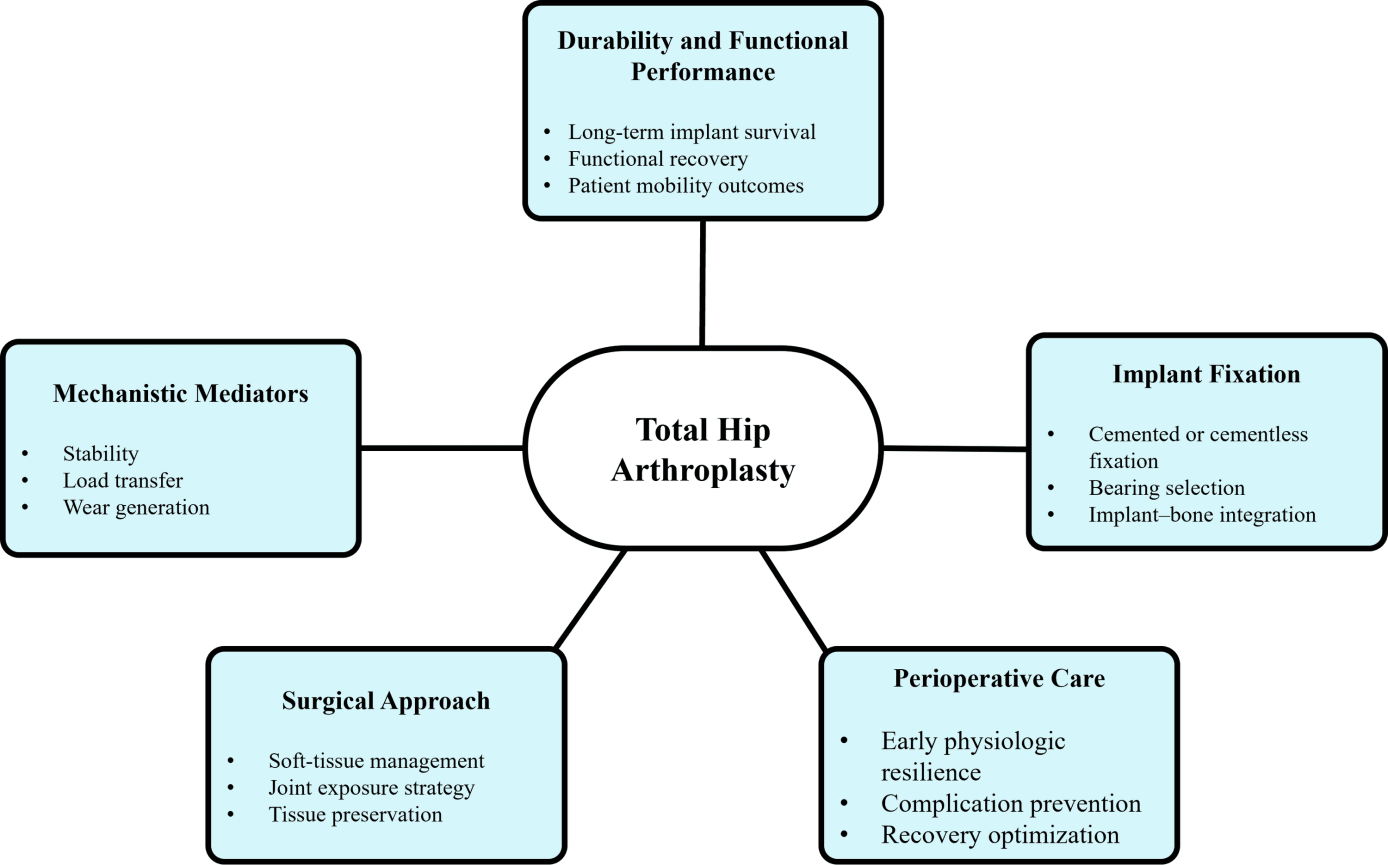
Integrated system of total hip arthroplasty. Created by the authors using Microsoft PowerPoint.

From the applied standpoint, ensuring the biomechanical and biological bases makes it possible to rationally combine the surgical technique and implant design instead of blindly following empirical choice [22]. Surgeons are increasingly forced to individualize fixation strategy, stem geometry, and bearing choice to patient-specific mechanical environments instead of being reassured by generalized performance claims [2]. Biomechanical and biological principles are the conceptual substrate on which meaningful evaluation of contemporary advances in THA must be grounded to ensure long-lasting and patient-centered outcomes [25]. Figure 1 illustrates the relationship between surgical technique, implant design, and recovery mechanisms.

Preoperative planning and patient-specific risk stratification

Preoperative planning in THA has evolved from rudimentary templating of THA to a multidimensional process that incorporates anatomic reconstruction and functional biomechanics while challenging the surgeon to individually assess risk [32]. Accurate prediction of implant size, offset restoration, and limb length equality, therefore, is at the heart of achieving stable load transfer and minimal postoperative dissatisfaction associated with gait disturbance or perceived inequality [6]. Contemporary planning is moving toward minimizing and eliminating intraoperative variability by the use of three-dimensional imaging and digital templating [14]. Patient-specific factors contribute to a significant alteration in the effectiveness of any surgical or implant-based advancement, hence the importance of stratified risk assessment against standardized procedural algorithms [33]. Bone quality, obesity, neuromuscular disease, spinopelvic mobility, and expectations of activity during the healing phase of the process affect fixation choice, stem geometry, and stability, which is often more predominant than just the approach during the surgical approach [18]. Failure to exercise caution that these variables can have a significant effect on the outcomes has been associated with an increased incidence of instability, periprosthetic fracture, and early revision despite technically correct implantation [7].

The preoperative recognition of spinopelvic imbalance and sagittal stiffness has led to new thinking of acetabular orientation, and it can now be understood that diplacental goals in terms of static alignment can be an inadequate goal in terms of dynamic functional stability [23]. This has resulted in recent population-based “safe zone” changes for population-based planning to a paradigm of functional planning involving adaptable component positioning principles relative to patient-specific patterns of motion [11]. Such concepts are one example of planning as a translational bridge between biomechanics theory and surgical execution [29]. Methodological limits remain, as much of the planning literature is based on simulation or correlation from retrospective studies or short-term therapeutic endpoints of radiography [15]. Evidence of the effectiveness of advanced planning tools for reducing dislocation, wear, or revision has been limited and often confounded by surgeon experience and the simultaneous use of enabling technologies [10]. As a result, the added value of sophisticated planning approaches cannot be assumed without reference to the bigger context of the operative and perioperative setting [26].

**Figure 2:**
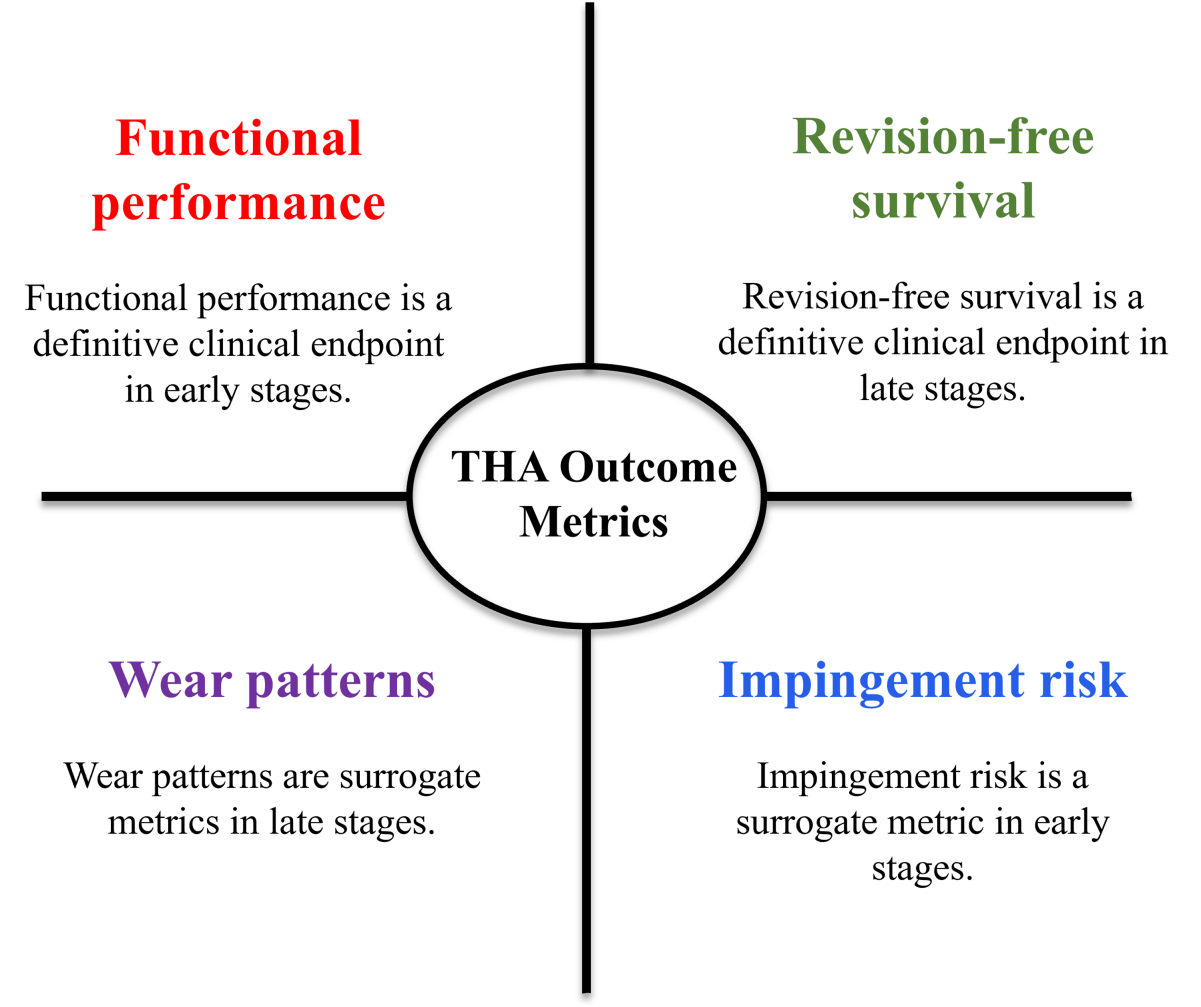
Component positioning and long-term construct pathway. Created by the authors using Microsoft PowerPoint. THA = total hip arthroplasty

From an applied perspective, good preoperative planning allows for the selective use of advanced implants, fixation strategies, and technologies where their mechanistic benefits are most likely to be converted into lasting benefit [34]. Risk stratification is important to support complex versus resilient patients and help implement outpatient pathways and enhance recovery protocols in the right population [12]. Preoperative planning is, therefore, not a separate preparatory step but a vital, integrative process unifying patient biology, implant mechanics, and surgical strategy to maximize the outcomes of the endless spectrum of THA indications [35]. Figure 2 indicates the pathway between component positioning and long-term construct behavior.

Surgical approaches and soft-tissue management strategies

Selection of the surgical approach in THA is a strategic decision that dictates exposure, soft-tissue preservation, and early functional recovery as opposed to a technical preference [36]. Posterior, direct anterior, and lateral or anterolateral approaches differ mainly in relationship with the capsular structures and abductor musculature, which also have differences in the influence on stability mechanics and whether these approaches affect rehabilitation trajectories [9]. Clinical outcomes associated with each approach are derived from the degree of successful surgical exposure that is balanced against preservation/restoration of stabilizing soft tissues [7]. From a mechanistic perspective, both surgical approaches used historically (among the posterior approaches) had a higher risk of dislocation, which is increasingly mitigated through meticulous technique of repair to the capsular structures and external rotator tendons [37]. Some techniques are biased toward intermuscular spaces and hypothetical muscle preservation but carry distinct challenges of femoral exposure, component placement, and limitations of learning curve-related issues [11]. Some approaches have the inherent stability (abductor splitting) affixed, and can impact the postoperative gait mechanics and perceived weakness of the patient for a longer time if soft-tissue healing is less than optimal [18].

Evidence often shows convergence of medium and long-term functional outcomes [15]. Despite early differences in pain results, gait, or length of stay, it is conceivable that the effects of the approaches may somehow diminish over time [38]. Such findings are often obtained from aggregated data, which masks effect modification by patient factors such as obesity, bone quality, or spinopelvic dynamics [3]. Soft-tissue management has become a uniting factor that is determining approach-related success, and re-focused debates end well away from incision place, toward capsular integrity, tension restoration, and avoidance of iatrogenic muscle damage [22]. Studies have increasingly found that structured capsular repair should be able to compensate orders of magnitude for the traditional risk of instability and to minimize the outcome differences between approaches [39]. This convergence recognizes that the surgical approach needs to be evaluated as a system of modifiable elements that include technique refinement and not considered as a risk category [16].

In applied practice, approach selection must be in line with surgeons’ expertise, patients’ anatomy, and the intended implant strategy to reduce avoidable complications [2]. High volume experience and consistency are more important than approach choice alone in determining reproducible outcomes [25]. Surgical approaches need to be understood as flexible models whose clinical effects are mediated by the quality of soft-tissue and patient selection beforehand and their integration with implant designs and recovery processes rather than as isolated determinants of clinical success [33]. Table 1 summarizes the mechanistic trade-offs between surgical approaches and soft-tissue strategies.

**Table 1:** Surgical approaches and soft-tissue strategies: mechanistic trade-offs. Original table created by the authors based on synthesis of the cited literature [9,18,36,38]. Therefore, no copyright permission or license agreement was required.

Surgical approach	Key soft-tissue interaction	Mechanistic advantage	Dominant risk profile	Representative reference
Posterior approach	Detachment and repair of the short external rotators and capsule	Excellent femoral exposure; flexible implant positioning	Instability without meticulous capsular repair	[36]
Direct anterior approach	Intermuscular, internervous plane	Muscle preservation; early gait normalization	Femoral exposure difficulty; fracture risk	[38]
Anterolateral approach	Partial abductor split	Intrinsic stability; low dislocation rates	Abductor weakness; gait alteration	[9]
Lateral approach	Direct abductor detachment	Wide exposure; stability	Persistent limp if healing is impaired	[18]

Component positioning and technology-assisted arthroplasty

Accurate component positioning is a central determinant of THA performance, as acetabular and femoral positioning affects joint stability, range of motion, wear patterns, and implant longevity [40]. Malpositioned components also run the risk of impingement, dislocation, edge loading, and accelerated wear of the polyethene component despite the use of modern bearing materials [7]. In addition to bearing-surface wear, component malposition can alter hip biomechanics by increasing offset-related loads, impingement forces, and bending moments across the head-neck taper. These abnormal mechanical stresses may contribute to mechanochemically assisted crevice corrosion at the trunnion, with subsequent metal ion release, adverse local tissue reactions, and trunnionosis-related failure [2]. These relationships have increased the level of accuracy of alignment from the technical detail to the fundamental mechanistic target of the relationship between the surgical execution and the long-term construct behavior [29].

Technology-assisted arthroplasty, including navigation, robotics, and intraoperative imaging of the implant, has been developed to deal with the inherent variability in freehand implantation and increase the reproducibility of planned alignment goals [41]. From a mechanistic point of view, these tools try to minimize the distribution of the component orientation, not redefine the best possible target; it is aimed at consistency, not absolute innovation [11]. Improved accuracy and reduced rates of outliers could be proven repeatedly, particularly for acetabular inclination and anteversion [14].

Positioning accuracy and clinical benefit are complex to bring about methodological translation. Several studies report surrogate endpoints such as precision of alignment or lower variance, but fewer studies report clear results of reduced dislocation, lower rates of wear failure, trunnion-related complications, or lower rates of revision at meaningful follow-up intervals [15]. This connection indicates that component positioning should not be interpreted only through surrogate radiographic endpoints, such as inclination, anteversion, or reduced alignment variance, but also through its downstream effect on modular-junction mechanics, trunnion bending moments, mechanochemically assisted crevice corrosion, and trunnionosis-related failure. Evidence shows that the accuracy of the alignment may have its most important clinical impact in high-risk populations, including abnormal spinopelvic mechanics or increased instability risk [23].

There are also learning curves, changes in workflow, and costs associated with robotic and navigated systems, which complicate the interpretation of outcomes [42]. Reported benefits are often based on high-volume centers or the early adopters, restricting the ability to generalize and making the assessment of incremental value over optimized conventional techniques difficult [16]. As technology use often accompanies good planning, standardization of pathways, and surgeon specialization, it is difficult to isolate independent effects [3].

From the applied point of view, technology-assisted positioning should be considered a risk modification strategy, rather than a requirement for everyone [2]. Selective use in anatomically complex situations, unstable patients, or in surgeons early in their experience may provide disproportionate benefit compared with routine use in low-risk situations [25]. Crucially, better alignment is no substitute for poor soft-tissue management, implant choice, or postoperative practices, and this supports the need for an integrative evaluation [22]. Component positioning technologies remove the emphasis of arthroplasty from the individual technical application and emphasize system reliability [43]. Their clinical relevance is dependent on the concurrence of mechanistic accuracy to patient-specific biomechanics and steadier goals of the result rather than individual steps of precision.

Implant fixation and structural design considerations

Fixation Strategy and Host-Bone Interaction

Implant fixation strategy and structural design are the mechanical backbone of THA, which determines initial stability, long-term load transfer properties, and predisposition to failure under physiologic stress [28]. The selection between cemented, cementless, and hybrid fixation is accordingly a combination of interactions involving implant geometry, surface technology, and quality of the host bone, instead of one superior construct [6]. As indications spread from elderly patients with compromised bone stock to younger patients with increased activity demands, fixation strategy is becoming increasingly decisive in determining both early complication risk and long-term survivorship patterns [28].

Biologic Osseointegration and Cemented Interfaces

From a mechanistic point of view, cemented fixation relies on the integrity of the mantle and the interdigitation of the cement and bone to distribute the load predictably, whereas cementless fixation relies on meeting the initial criteria of press-fit stability followed by biologic osseointegration [28]. Micromotion above critical limits hinders bone ingrowth, whereas excessive stiffness of the construct may cause stress shielding and proximal bone resorption; therefore, the balance of structural behavior is crucial [11].

Porous Titanium and Additive Manufacturing

Advances in porous coatings, highly porous titanium, and additive manufacturing are geared toward optimization of this balance by promoting rapid and durable bone surface on-growth or in-growth [31]. Recent 3D-printed highly porous titanium designs extend this principle by using biomimetic trabecular architectures intended to increase pore interconnectivity, reduce stiffness mismatch, and support cementless osseointegration; however, longer-term survivorship data are still needed before these technologies can be considered superior to established cementless fixation surfaces [31].

Stem Geometry and Load-Transfer Dynamics

Femoral stem geometry also has an additional modulatory effect on the success of the prosthesis fixation because of changes in the pattern of load transfer and risk of fracture [29]. Metaphyseal-engaging and tapered wedge stems have their advantages of proximal load sharing, whereas shorter stems have the advantage of bone preservation but may introduce sensitivity to surgical technique and patient anatomy [29,30]. Studies suggest an important interaction between stem design, surgical approach, and bone quality, especially in terms of the risk of early periprosthetic fracture [29].

Evidence Limitations and Patient-Specific Fixation

A great deal of fixation literature comes from registry analysis and retrospective cohorts that are useful for capturing signals for revision and complications at scale, but provide less insight into variables in the operating theater and rehabilitation context [28]. Comparisons between fixation strategies are often confounded by age, indication, and regional patterns of practice, which makes causal inference difficult [28]. Short- and mid-term follow-up further limits the evaluation of whether newer surface technologies have any meaningful impact on long-term trajectories of loosening or revision [31]. In applied practice, the choice of fixation strategy is becoming an individualized process, less dependent on institutional convention and more closely aligned with patient-specific needs [28]. Older patients with osteoporotic bone may benefit from cemented fixation in terms of early safety, whereas younger patients with good bone quality may benefit from biologic fixation to preserve future revision options [28]. Structural design choices, therefore, have to be integrated with surgical approach, alignment strategy, and postoperative loading environment to minimize trade-offs across the life cycle of the implant [44]. Implant fixation and design advances should be interpreted with respect to their ability to balance mechanical demands and biologic response over time, rather than being judged only by isolated survivorship measures [28,31]. Such an understanding aligns structural innovation with the wider system of care determining durable success in contemporary THA. Table 2 shows the effect of fixation and implant design on load transfer and durability.

**Table 2:** Implant fixation and structural design: biomechanical-biological integration. Original table created by the authors based on a synthesis of the cited literature [34,45-47]. Therefore, no copyright permission or license agreement was required.

Design variable	Mechanistic role	Clinical benefit	Principal trade-off	Representative reference
Cemented fixation	Uniform load transfer via cement mantle	Reduced early fracture risk in osteoporotic bone	Cement-related failure modes	[45]
Cementless porous fixation	Osseointegration through bone ingrowth	Long-term biologic fixation	Early micromotion sensitivity	[46]
Tapered wedge stem	Proximal metaphyseal loading	Bone preservation; physiologic load	Technique-sensitive positioning	[47]
Short femoral stem	Reduced distal fixation	Bone stock preservation	Subsidence risk in poor bone	[34]

Bearing surfaces, head-neck interfaces, and stability constructs

Bearing surface choice/head-neck interface configuration of THA plays a central role in mediating the wear behavior and the stability mechanics and long-term biological response [13]. The development of conventional polyethene to highly cross-linked polyethene was an important step in the development of the clinical presentation of polyethene by dramatically slowing the rate of volumetric wear and osteolysis that leads to prolonged implant survivorship, particularly in younger and more active patients [31]. However, reduced wear alone is not a guarantee of long-term success of these bearings as performance still depends on the accuracy of the alignment, size of the head, and loading conditions [40]. Ceramic heads have been introduced to further reduce wear and oxidative deterioration at the articulating surface and have better hardness and scratch resistance than metal counterparts [19]. Their clinical benefit is most obvious when cross-linked with highly cross-linked polyethene, but clinical benefits may be diminished due to malposition, impingement, or excessive activity profiles [7].

Ceramic-on-ceramic constructs have excellent wear characteristics with peculiar risks of squeaking and fracture; therefore, only selected populations can be considered for this approach [11]. Head size selection and real stability computability are trade-offs inherent in stability-oriented design. Larger femoral heads enhance jump distance and range of movement before impingement, which decreases the risks of dislocation in many settings [28]. When the head diameter is increased, stresses at the taper junction increase, and mechanically assisted crevice corrosion is assessed as a problem, especially as seen with modular constructs [5]. These opposing mechanisms highlight the need for stability capabilities to be balanced with that of interface durability as opposed to unconditionally pursued [44]. Dual mobility systems are a good example of an integrative approach to instability prevention by using a combination of a mobile polyethene liner with a large effective head diameter [48]. Such constructs have been shown to increase the range of stable function and to have decreased rates of dislocation in high-risk patients, including neuromuscular patients or patients with revision risk factors [23]. Unique forms of failure, such as intraprosthetic dislocation and issues with respect to long-term wear, are added processes to their wider use [16].

From an applied standpoint, bearing and head-neck choices should be tailored to each individual based on the age, activity, risk of instability, and configuration of the implant and not reinforced or standardized to different populations [2]. Effective integration of bearing technology with component positioning, soft-tissue management, and fixation strategy is still required to provide desired benefits [33]. Accordingly, bearing surfaces and stability constructs should be viewed as adaptive tools within a larger mechanical and biologic system that specifies the in-time performance of THA [27].

Perioperative pathways and early recovery optimization

Perioperative management has become an important factor in the outcome of THA due to its influence on physiologic stress, early mobilization, and risk of complications, rather than being just a supportive adjunct to surgery [12]. Enhanced recovery after surgery pathways utilize a combination of multimodal analgesia, blood conservation strategies, standardized anesthesia, and early rehabilitation to shorten the time frames and keep it safe [41]. These protocols reflect a paradigm shift in the mechanistic approach to limit inflammatory response and opioid exposure and immobilization, and affect both the short-term outcome and downstream utilization [3]. From a biological perspective, optimized pain control and early mobilization reduce neuroendocrine stress and aid in functional recovery to indirectly reduce risks, such as thromboembolism and delirium, as well as early instability [28]. Tranexamic acid is a good example of a pharmacologic intervention because it decreases blood loss during surgery without putting patients at risk for thromboembolism, allowing for more rapid mobilization and readiness to discharge [45]. Such measures are illustrative of the manipulations that can be made to the recovery environment by perioperative interventions that can interact with surgical technique and implant stability [22].

A substantial body of evidence from systematic reviews and cohort studies indicates that enhanced recovery pathways are associated with reduced length of stay without an increase in complication rates compared with conventional care [31]. However, pathway components vary widely between institutions, which limits the ability to attribute observed benefits to specific elements and reduces reproducibility [15]. Early recovery outcomes (length of stay or same-day discharge) may not be a good predictor of longer-term functional outcome and patient satisfaction [17]. The expansion of outpatient and short-stay THA has led to the applied relevance of optimization of the perioperative process [48]. Successful implementation requires much from the selection of patients, social support, and integration with surgical and anesthetic strategies rather than pathway protocols alone [9]. Studies with reports of positive outcomes of outpatient treatment are often from high-volume centers with well-built infrastructure, raising questions of scalability and equity [16]. Accelerated recovery does not mean that vigilance is not required in terms of complications, such as falls and early dislocation/wound problems, especially in elderly or high-risk populations [18]. This brings to attention that perioperative pathways are force multipliers with safety and effectiveness dependent on congruence with patient-specific risk profiles [2].

From an integrative perspective, perioperative optimization should be evaluated as part of the THA system, and not as a separate intervention [27]. Its greatest value is to add to the benefits of good surgical execution and appropriate selection of implant rather than compensate for deficiencies in either area [44]. Early recovery pathways should be interpreted in a far-reaching way that includes balancing between efficiency and longevity, patient safety, and long-term functional success [33]. Table 3 shows the critical components of the perioperative pathways and early effects of recovery.

**Table 3:** Perioperative pathways and early recovery: system-level effects. THA = total hip arthroplasty Created by the authors based on information synthesized from previously published studies [12,31,45,48]. Therefore, no copyright permission or license agreement was required.

Pathway component	Targeted mechanism	Outcome influence	Implementation dependency	Representative reference
Multimodal analgesia	Reduced central sensitization	Early mobilization; opioid reduction	Protocol adherence	[12]
Tranexamic acid	Inhibition of fibrinolysis	Reduced blood loss; faster recovery	Timing and dosing	[45]
Early mobilization	Neuroendocrine stress reduction	Lower thromboembolic risk	Physical therapy access	[31]
Outpatient THA protocols	Care compression	Reduced length of stay	Patient selection and support	[48]

**Figure 3:**
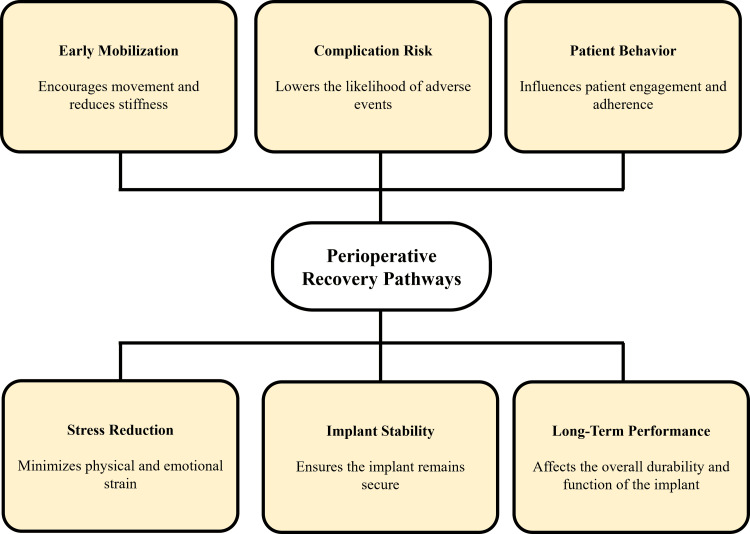
Recovery trajectories and functional durability. Created by the authors using Microsoft PowerPoint.

Figure 3 demonstrates how recovery trajectories interact with long-term functional performance.

Durability, functional performance, and long-term construct behavior

Given the need for long-term therapy, it is important to recognize that long-term success in THA can be determined by survival of the implant-host construct and long-term capacity to maintain functional performance in the face of cumulative mechanical and biological stress and not just by early postoperative milestones [27]. Implant survivorship, revision-free survival, and maintenance of patient-reported functional gains are the epigram functions of surgical technique, implant design, and sanitary loading environments over time [15]. Such outcomes are integrative endpoints in which early decisions have late consequences [44]. From a mechanistic point of view, the long-term behavior of a construct is regulated by the combination of fixation stability, wear generation, and the host-bone adaptation [31]. Subclinical micromotion, low-grade wear particle release, or gradual stress shielding may not manifest for years and may only become manifest by the loosening, osteolysis, or mechanical failure [49]. Durability is less of a stable attribute of implant materials but more of an emergent attribute of system balance in biologic and mechanical domains [22].

Functional performance is another aspect of understanding long-term success in which patient experience of construct behavior during daily and high-demand activities [17]. Sustained improvements in gait and mobility and tolerance of activities are dependent upon restoration of biomechanics, preservation of muscle function, and avoidance of chronic pain generators such as impingement or instability [29]. There is evidence that acceptable implant survivorship does not equate uniformly to ideal outcomes of function, especially in younger age groups and/or high-demand cohorts [3]. Assessment of durability and function is limited by reliance on revision as an endpoint, which may underestimate suboptimal performance that does not result in reoperation [50]. Patient-reported outcome measures provide additional information, but their collection and interpretation are variable, and long-term follow-up beyond mid-term time points is often lacking [34]. These factors limit the ability to synthesize how specific surgical or implant-related developments influence perceptions of sustained quality of life [16].

Applied interpretation needs to acknowledge that long-term performance is sensitive to cumulative exposure as opposed to occasion-based, resulting in surgical success. Younger patients with higher levels of activity may impose more burden on bearing surfaces and fixation interfaces, whereas the older age group may experience functional decline due to comorbidities rather than implant failure [18]. Such is the heterogeneity of the subject, the need for the individualization of expectations, and strategies of longitudinal monitoring [25]. In an integrative sense, durability and functional performance constitutes the convergence point of all previously separate areas within THA [40]. They provide the most clinically meaningful test of whether the improvements in technique, technology, and pathways meet the intended purpose over the entire life cycle of the implant [49].

Limitations and future directions

Some limitations limit the understanding of the contemporary THA literature. Evidence is often fragmented between surgical technique, implant design, and perioperative management, as systems-level inference is limited. Heterogeneity in study design, choice of patients, duration of follow-up, and outcome reporting weakens comparability and invalidates the synthesis. Numerous investigations are focusing on short-term or surrogate endpoints as opposed to long-term reliable function and risk for revision. Rapid adoption of technology is also followed by long-term validation of technology, with uncertainty about long-term clinical value in diverse patient groups globally.

Future research should prioritize the use of integrative study designs that compare techniques, implants, and recovery pathways at the same time. Standardized reporting of patient-reported outcomes, functional metrics, and complication definitions is important to enable robust synthesis to be done. Longer follow-ups are necessary as the durability of newer materials, fixation strategies, and technologies needs to be determined. These issues of internal validity and relevance to the real world might be addressed by pragmatic randomizsed trials and registry-based trials. Strategies that emphasize patient-specific stratification, value assessment, and implementation science aid the translation of innovation to consistent and equitable clinical benefit across health systems and surgical settings internationally and across time periods.

## Conclusions

This review brings together some of the latest developments in THA from a multifaceted perspective, combining the technical aspects of surgery, implant design, and perioperative care within a single analytical framework. These aspects suggest that successful outcomes are not achieved by single innovations, but by a combination of coordinated optimizations of biomechanics, biological fixation, and recovery environments. All of these surgical techniques, i.e., positioning, fixation, and bearing selection, have the same mechanism of action and have a cumulative effect on implant stability, wear, function, and longevity. These relationships are further modulated by enhanced recovery pathways, affecting early mobilization and physiologic resilience. Although each step might seem small or unexpected at a given time when examined alone, together, they can provide a clear pattern to explain trade-offs and context dependencies. The review highlights the importance of considering patient-specific factors as they greatly influence outcomes; thus, individual decision-making will be needed instead of a one-size-fits-all approach to the adoption of technologies or techniques. The integrative evaluation allows the translation of the plausibility of the mechanism of action into clinically relevant benefit without inflation of surrogate endpoints. In the long-term, viewing THA as an engineered clinical system makes rational innovation, prediction of outcomes, and integration of future research with patient-centered durability possible.
